# Breastfeeding Practices and Associated Factors Among Mothers of Infants Aged 12 Months or Younger Attending Selected Tertiary Hospital in Dhaka, Bangladesh

**DOI:** 10.1002/fsn3.71323

**Published:** 2025-12-04

**Authors:** Dilshad Binta Zaman Disha, Md. Shamsur Rahman, Munir Ibn Mahin

**Affiliations:** ^1^ Department of Nutrition and Food Engineering Daffodil International University Savar Dhaka Bangladesh

**Keywords:** Bangladesh, exclusive breastfeeding, infant feeding, level practice, maternal factors, sociodemographic determinants

## Abstract

Breastfeeding is essential for optimal infant growth and development which promotes health by preventing malnutrition and reducing the risk of infectious diseases. This study aimed to assess breastfeeding practices among mothers and identify factors influencing these practices among mothers of infants aged 12 months or younger in a tertiary hospital in Dhaka, Bangladesh. A cross‐sectional survey was conducted from June to August 2023 at Dhaka Medical College Hospital (DMCH). A total of 250 mothers with infants aged ≤ 12 months were recruited using Cochran's formula. Data were collected through a structured, pre‐tested questionnaire via face‐to‐face interviews. Data were analyzed using SPSS version 27, chi‐squared tests and multivariable logistic regression analyses were performed (*p* < 0.05). The prevalence of exclusive breastfeeding (EBF) was 80.4%. In adjusted models, mothers aged ≤ 26 years were less likely to exclusively breastfeed compared to older mothers (AOR = 0.58, 95% CI: 0.44–0.78, *p* < 0.001). Mothers with primary education also showed reduced odds of EBF compared to those with no education (AOR = 0.67, 95% CI: 0.48–0.94, *p* = 0.020). By contrast, higher breastfeeding practice scores were positively associated with EBF (AOR = 1.30, 95% CI: 1.16–1.45, *p* < 0.001). Other sociodemographic factors, including infant age, birth order, father's education, maternal occupation, place of delivery, and infant sex, were not significant in the multivariable analysis. Breastfeeding practices in this population were strongly influenced by maternal age, maternal education, and breastfeeding practice levels. Targeted interventions focusing on young and less‐educated mothers, alongside improved breastfeeding support, may further strengthen EBF practices and improve infant health outcomes in Bangladesh.

## Graphical abstract

This cross‐sectional study assessed breastfeeding practices and associated factors among 250 mothers in Dhaka, Bangladesh. Exclusive breastfeeding prevalence was 80.4%. Younger mothers (≤ 26 years) and those with only primary education were less likely to exclusively breastfeed, while higher breastfeeding practice scores increased likelihood. The findings highlight the need for enhanced support and education for younger and less‐educated mothers to sustain exclusive breastfeeding and improve infant health outcomes.

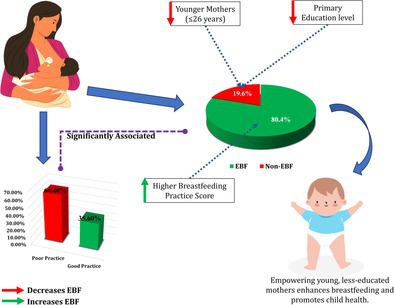

## 1 Introduction

Breastfeeding is a critical determinant of infant survival and development, especially in low‐ and middle‐income countries (Mallick et al. 2021). Breast milk provides balanced and easily digestible nutrients, supports optimal growth, and protects against infections and malnutrition (Sánchez et al. 2021). It also reduces the risk of gastrointestinal and respiratory illnesses, sudden infant death syndrome, and later‐life conditions such as diabetes and certain cancers (Alotiby 2023; Capra et al. 2025; Froń and Orczyk‐Pawiłowicz 2024; Kariyawasam et al. 2025). For mothers, breastfeeding decreases the risk of postpartum hemorrhage, breast and ovarian cancers, type 2 diabetes, and contributes to natural birth spacing through lactational amenorrhea (Alimi et al. 2022; Stordal 2023; Wang et al. 2021).

Despite these benefits, exclusive breastfeeding (EBF) rates remain suboptimal worldwide (Miatton et al. 2025). Only about 44% of newborns receive breastmilk within one hour of birth, and just 40% of infants are exclusively breastfed for the first six months (Victora et al. 2016). Global analyses show that appropriate breastfeeding coverage could reduce under‐five mortality by up to 15.5% (Kefale et al. 2025). However, many children continue to be bottle‐fed or inadequately breastfed during this crucial period (Garti et al. 2023).

In South Asia, prevalence varies widely: India reports 55% EBF, Nepal 66.1%, Bhutan 51.4%, and Sri Lanka 70% (Bhandari et al. 2019; Ogbo et al. 2019; Perera et al. 2012; Pokhrel et al. 2018; Wasti et al. 2023). Bangladesh, despite recent improvements, still faces challenges. The rate of exclusive breastfeeding was 63.4% in 2019 (Hasan et al. 2021), yet malnutrition persists, with more than half of children under five classified as underweight (Hossain et al. 2018). Early initiation of breastfeeding, which is known to improve the duration of breastfeeding and maternal outcomes, remains inconsistently practiced (Abdulahi et al. 2021; Hassen 2024).

Although several global and regional studies highlight the determinants of breastfeeding (Shi et al. 2021; Standish and Parker 2022; Tigka et al. 2022; Woldeamanuel 2020), evidence specific to Bangladesh remains limited. Existing studies often suffer from methodological weaknesses, such as small sample sizes or inadequate statistical analyses, leaving uncertainty about the influence of maternal and sociodemographic factors on breastfeeding practices.

Therefore, the present study aims to determine the prevalence of breastfeeding practices among mothers of infants aged 12 months or younger and to identify the factors that influence breastfeeding practices in tertiary hospitals in Dhaka, Bangladesh.

## 2 Methods

### 2.1 Study Design

This cross‐sectional study was conducted from June to August 2023 at Dhaka Medical College Hospital, Secretariat Rd., Dhaka‐1000, Bangladesh.

### 2.2 Settings

Dhaka, the capital city of Bangladesh, has a population of approximately 175.7 million (17.6 crore). Dhaka Medical College Hospital (DMCH) was selected as the study location. Because, it is one of the biggest, oldest, and most established medical colleges/hospitals in Bangladesh. Additionally, it is a government hospital and the fees are generally lower compared to other private hospitals in Dhaka, making it easily accessible for extensive maternal and child healthcare services which attract mothers from diverse socioeconomic backgrounds. The study included mothers attending the outpatient department to receive maternal and child healthcare services.

### 2.3 Sample Size Determination

The required sample size was calculated using Cochran's formula:

$$\text{Sample size},n=\frac{Z^{2}\mathrm{pq}}{e^{2}}$$

*Z*: 1.96 at a 95% confidence level, *p*: prevalence of exclusive breastfeeding was 63.4% (Hasan et al. 2021) *q* = 1‐*p*, *ε*: margin of error (5.98%). A total of 250 mothers were chosen to participate in the research based on the estimated sample size.

### 2.4 Data Collection Tools

A structured questionnaire was developed based on prior literature and the study objectives. Items were adapted from a previously validated study on breastfeeding practices (Agunbiade and Ogunleye 2012). The questionnaire was refined through academic expert review and pre‐testing. It was presented to each mother with a clear explanation of the study's objective. A Bengali‐translated version was used in the study.

Both closed‐ended and open‐ended questions were included in the survey. Before being utilized for data collection, the questionnaire was pre‐tested with 20 participants to measure understanding, readability, and administration convenience. The questionnaire includes a variety of questions across several sections to capture a comprehensive view of the participants' demographics, breastfeeding practices, and the challenges they face with breastfeeding practices. The first section focuses on the demographic characteristics of the mothers and infants. These questions aim to understand the participants' basic demographic profiles, age of infants and mothers, birth order of infants, education level of mother and father, occupation of mother, place of delivery, gender of the baby. The second section assesses Breastfeeding Practices which assessed the practice by following questions related to the initiation of breastfeeding, colostrum feeding, breastfeeding information, frequency of breastfeeding, duration of breastfeeding sessions, use of supplements, and intended duration of breastfeeding related questions were part of the second section.

The scoring was done according to the methods described by (Agunbiade and Ogunleye 2012). Responses to each question scored 0–5. Breastfeeding practice level was scored on a scale of 100%; if the score is 0%–50%, it indicates poor practice; if the score is 51%–100%, it indicates good practice. Exclusive breastfeeding practices were also categorized into (a) Yes (≤ 6 months of breastfeeding), and (b) No (> 6 months to ≤ 12 months of breastfeeding). The practice‐level score excluded the question defining exclusive breastfeeding to ensure independence between predictor and outcome variables.

### 2.5 Data Collection

The authors addressed mothers and babies seeking treatment at Dhaka Medical College Hospital. The study's goals and objectives, as well as the rewards and hazards of participation, were clearly articulated to the mothers to encourage their active involvement and collaboration. Data collection took around 10 min and included face‐to‐face interviews with mothers who chose to take part in the study.

### 2.6 Inclusion and Exclusion Criteria

**FIGURE 1:**
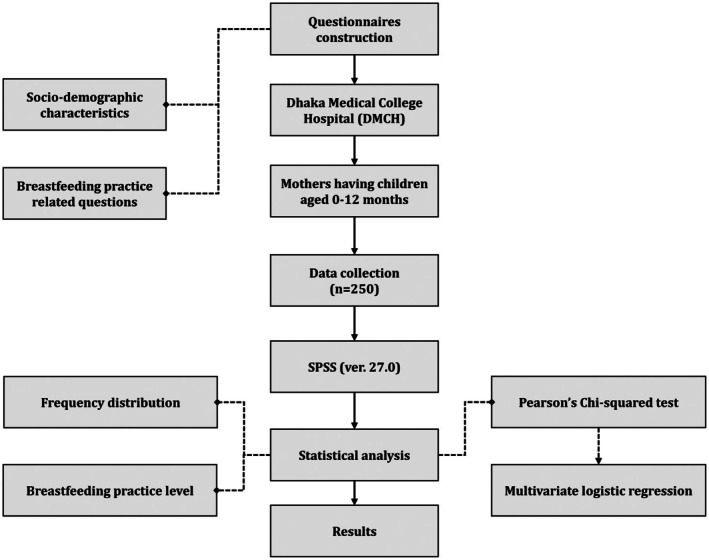
Flow diagram of the study.

Mothers were eligible for this research if they (a) had children aged 12 months or younger, (b) were mentally competent and capable of verbal communication, (c) were physically present during data collection, and (d) consented to participate willingly. Mothers were excluded if they (a) had medical illnesses or drugs that hindered breastfeeding, (b) were diagnosed with severe congenital abnormalities, (c) had children older than 12 months, (d) had babies with any acute or chronic disease, or (e) were HIV/AIDS positive. Figure 1 represents the flow of participant selection and statistical analysis.

### 2.7 Statistical Analysis

Data analysis was conducted using SPSS software (version 27.0) for Windows (Armonk 2020). Descriptive statistics were used to present socio‐demographic factors affecting breastfeeding. Continuous variables were summarized as means ± SD, and categorical variables as frequencies and percentages. The total practice ratings were transformed to a percentage of 100% (Agunbiade and Ogunleye 2012; FAO/OMS 2014). Univariate associations were assessed using Pearson's chi‐square testing. A multivariate logistic regression model was created utilizing the important parameters from the univariate test to identify independent determinants of breastfeeding habits among women. *p*‐values < 0.05 were considered statistically significant.

### 2.8 Ethical Approval

The research protocol was reviewed and approved by the Institutional Review Board, Daffodil International University (Ref: FAHSREC/DIU/2023SMIG‐33) before the study began. Furthermore, throughout the entire process all national and international ethical standards for research involving human beings were followed and applied.

## 3 Results

Table 1 presents the sociodemographic and breastfeeding characteristics of mothers in this study (*n* = 250). Among the infants, 71.6% were aged ≤ 6 months, while the remaining 28.4% were between > 6 and < 12 months. Most mothers (70.8%) were aged ≤ 26 years, with a larger proportion of exclusive breastfeeding observed in this group. In terms of birth order, two‐thirds (66.4%) of the infants were of birth order ≤ 2. Regarding educational attainment, 18.8% of mothers had no education, 58.4% had completed primary education, and 22.8% had secondary or tertiary education. The fathers showed a similar distribution, with 16.8% having no formal education, nearly half (47.6%) having primary education, and 35.6% achieving secondary or tertiary education. The majority of mothers (57.6%) worked in industrial labor, while 28.8% were housewives, and 13.6% were employed in services. Delivery settings showed that 82.0% of the births occurred in public hospitals, with a smaller proportion in private hospitals (14.8%) or under traditional birth attendants (3.2%). Finally, regarding the gender of the infant, 43.2% were male and 56.8% female. Pearson's chi‐squared test indicated significant associations between several sociodemographic characteristics and exclusive breastfeeding, with *p*‐values < 0.05 for age, education, occupation, delivery location, and gender.

**TABLE 1:** Socio‐demographic characteristics of study participants (*n* = 250).

Characteristics	Total	Exclusive breastfeeding		*χ* ^2^	*p* value
		Yes	No		
		201 (80.4%)	49 (19.6%)		
Age of infants					
≤ 6 months	179 (71.6%)	157 (62.8%)	22 (8.8%)	21.370	< 0.001*
> 6 months to < 12 months	71 (28.4%)	44 (17.6%)	27 (10.8%)		
Age of mothers					
≤ 26 years old	177 (70.8%)	163 (65.2%)	14 (5.6%)	52.570	< 0.001*
> 26 years old	73 (29.2%)	38 (15.2%)	35 (14.0%)		
Birth order of infants					
≤ 2	166 (66.4%)	152 (60.8%)	14 (5.6%)	39.091	< 0.001*
> 2	84 (33.6%)	49 (19.6%)	35 (14.0%)		
Education level of mother					
No education	47 (18.8%)	20 (8.0%)	27 (10.8%)	74.249	< 0.001*
Primary	146 (58.4%)	142 (56.8%)	4 (1.6%)		
Secondary or tertiary	57 (22.8%)	39 (15.6%)	18 (7.2%)		
Education level of father					
No education	42 (16.8%)	19 (7.6%)	23 (9.2%)	43.907	< 0.001*
Primary	119 (47.6%)	110 (44.0%)	9 (3.6%)		
Secondary or tertiary	89 (35.6%)	72 (28.8%)	17 (6.8%)		
Occupation of mother					
Housewife	72 (28.8%)	58 (23.2%)	14 (5.6%)	24.547	< 0.001*
Service holder	34 (13.6%)	17 (6.8%)	17 (6.8%)		
Labor work in Industry	144 (57.6%)	126 (50.4%)	18 (7.2%)		
Place of delivery					
Public hospital	205 (82.0%)	173 (69.2%)	32 (12.8%)	11.508	0.003*
Private hospital	37 (14.8%)	23 (9.2%)	14 (5.6%)		
Traditional birth attendant/mission homes	8 (3.2%)	5 (2.0%)	3 (1.2%)		
Gender of the baby					
Male	108 (43.2%)	78 (31.2%)	30 (12.0%)	8.069	0.005*
Female	142 (56.8%)	123 (49.2%)	19 (7.6%)		

Abbreviation: *χ* ^2^, Pearson's chi‐squared.

* Statistically significant: *p* < 0.05.

The association of practice level with exclusive breastfeeding is shown in Table 2. Among the 250 mothers, 80.4% practiced exclusive breastfeeding. The majority (64.4%) had a poor practice level, with 60.8% of these mothers exclusively breastfeeding. Conversely, 35.6% of mothers demonstrated good practice levels, of which 19.6% practiced exclusive breastfeeding and 16.0% practiced normal breastfeeding. A chi‐squared test revealed a statistically significant association between practice level and breastfeeding type (*χ* ^2^ = 56.330, *p* < 0.001), indicating that practice level was closely related to breastfeeding practices among mothers in this study.

**TABLE 2:** The association of practice level with exclusive breastfeeding (*n* = 250).

Characteristics	Total	Exclusive breastfeeding		*χ* ^2^	*p*
		Yes	No		
		201 (80.4%)	49 (19.6%)		
Practice level					
Poor practice	161 (64.4%)	152 (60.8%)	9 (3.6%)	56.330	< 0.001*
Good practice	89 (35.6%)	49 (19.6%)	40 (16.0%)		

* Statistically significant: *p* < 0.05.

Table 3 presents the results of a multiple logistic regression analysis examining factors associated with exclusive breastfeeding. Several variables showed significant associations. Mothers aged ≤ 26 years had significantly lower odds of exclusive breastfeeding compared to those older than 26 years (AOR = 0.58; 95% CI: 0.44–0.78; *p* < 0.001). Similarly, mothers with a primary education level had reduced odds of exclusive breastfeeding compared to mothers with no education (AOR = 0.67; 95% CI: 0.48–0.94; *p* = 0.020). Breastfeeding practice level was strongly associated with exclusive breastfeeding: mothers with poor practice scores were much less likely to exclusively breastfeed (AOR = 0.24; 95% CI: 0.20–0.29; *p* < 0.001) compared to those with good practice scores. Other predictors, including infant age, birth order, father's education, maternal occupation, place of delivery, and infant sex, did not demonstrate statistically significant associations with exclusive breastfeeding in the adjusted model (*p* > 0.05). Model fit indices indicated that the logistic regression explained a meaningful proportion of the variance in exclusive breastfeeding, with good calibration and discrimination (Nagelkerke *R* ^2^ = 0.28; Hosmer–Lemeshow *χ* ^2^ (8) = 6.21, *p* = 0.62; AUC = 0.79).

**TABLE 3:** Factors affecting exclusive breastfeeding practice.

Predictor	AOR	95% CI		*p*
		LL	UL	
Age of infants (Ref. < 6 months)				
> 6 months to < 12 months	1.01	0.81	1.28	0.906
Age of mothers (Ref. > 26 years old)				
< 26 years old	0.58	0.44	0.78	**< 0.001** *
Birth order of infants (Ref. > 2)				
< 2	0.81	0.62	1.05	0.109
Education level of mother (Ref. no education)				
Primary	0.67	0.48	0.94	**0.020** *
Secondary or tertiary	1.28	0.94	1.73	0.118
Education level of father (Ref. no education)				
Primary	1.17	0.84	1.64	0.350
Secondary or tertiary	1.04	0.75	1.43	0.816
Occupation of mother (Ref. service holder)				
Housewife	0.76	0.53	1.09	0.131
Labor work in industry	0.74	0.52	1.07	0.112
Place of delivery (Ref. traditional birth attendant)				
Public hospital	0.61	0.36	1.05	0.073
Private hospital	0.67	0.38	1.19	0.168
Gender of the baby (Ref. male)				
Female	0.93	0.77	1.11	0.420
Breastfeeding practice level (Ref. good practice)				
Poor practice	0.24	0.20	0.29	**< 0.001** *

Abbreviations: AOR, adjusted odds ratio; CI, confidence interval; LL, lower limit; UL, upper limit.

*Note*: Bold values indicates *Statistically significant: *p* < 0.05.

Variance Inflation Factor values (< 2) indicated no major collinearity. Variables significant in bivariate analysis but not in the multivariate model likely reflect confounding by maternal age and education.

## 4 Discussion

This study examined exclusive breastfeeding (EBF) practices among mothers attending a large tertiary hospital in Dhaka, Bangladesh, and explored sociodemographic and maternal factors influencing these practices. The prevalence of EBF in our study population was 80.4%, a rate substantially higher than the national average of 65% (Hasan et al. 2021) and markedly greater than rates reported in previous studies, where only about one‐third of infants are exclusively breastfed (Hossain et al. 2018; Mohamed et al. 2018). The relatively high prevalence observed in this study reflects that these mothers have greater access to healthcare counseling and exposure to breastfeeding promotion initiatives.

The prevalence of EBF in this study is consistent with research conducted in other urban hospital contexts in South Asia, where access to trained health professionals and institutional deliveries provide an enabling environment for breastfeeding initiation and continuation. Studies from Nepal and Sri Lanka, for example, report comparably higher EBF prevalence in urban tertiary care hospitals than in rural regions (Bhandari et al. 2019; Perera et al. 2012). Conversely, the EBF rates in this study were notably higher than those reported in rural Bangladesh, where lack of access to trained healthcare providers, lower maternal literacy, and entrenched socio‐cultural practices such as pre‐lacteal feeding reduce adherence to WHO‐recommended breastfeeding practices (Hossain et al. 2018).

In multivariable analysis, maternal age was a strong and independent predictor of EBF. Mothers aged ≤ 26 years were significantly less likely to exclusively breastfeed compared to their older counterparts. This finding contrasts with some earlier studies that suggested younger mothers are more compliant with EBF due to greater exposure to health campaigns or antenatal counseling (Rahman et al. 2020). Instead, this study's results align with research indicating that younger mothers may face greater challenges in sustaining EBF because of competing social and economic responsibilities, limited experience with infant care, and increased vulnerability to peer or family pressures discouraging breastfeeding (Bengough et al. 2022; Bookhart et al. 2021; Grant et al. 2022; Norman et al. 2022).

Maternal education also emerged as an independent determinant of EBF. Mothers with only primary education had significantly lower odds of exclusive breastfeeding compared to those with no education. This result may appear counterintuitive, as higher educational attainment is often associated with better health knowledge and positive child‐feeding practices (Velusamy et al. 2017). However, this study's findings are consistent with a growing body of literature suggesting a complex relationship between maternal education and breastfeeding. While education may increase awareness of the benefits of breastfeeding, it also exposes mothers to alternative feeding practices such as formula feeding, especially in urban settings where commercial marketing of breastmilk substitutes is pervasive (Rollins et al. 2023). Moreover, primary education alone may not provide sufficient empowerment or access to resources that enable mothers to sustain EBF.

The breastfeeding practice score was one of the strongest predictors of EBF in this study. Mothers with higher practice scores, reflecting knowledge, attitudes, and reported behaviors consistent with recommended feeding practices were substantially more likely to exclusively breastfeed (Dukuzumuremyi et al. 2020; Gebeyehu et al. 2023). This association is consistent with studies from Kenya, Nigeria, and other low‐ and middle‐income countries, where maternal knowledge and confidence have been shown to directly influence breastfeeding duration and exclusivity (Akinyinka 2016; Mohamed et al. 2018).

Several factors that appeared significant in bivariate analysis, including infant age, paternal education, maternal occupation, place of delivery, and infant sex, were no longer associated with EBF in the adjusted model. This attenuation likely reflects confounding among sociodemographic variables. For example, maternal occupation is closely linked to both maternal age and education, which were retained as stronger predictors in the model (Yadav et al. 2021). Similarly, the initial significance of paternal education may have been mediated by maternal education, as household educational attainment is often correlated.

This study contributes valuable evidence on breastfeeding practices in an urban Bangladeshi setting, where facility‐based care and sociodemographic factors interact in complex ways. Strengths include the relatively large sample size, use of a structured and pre‐tested questionnaire, and application of multivariable regression to control for confounding.

However, several limitations should be acknowledged. First, the cross‐sectional design limits causal inference, as associations may not reflect temporal sequences. Second, reliance on maternal self‐report introduces the potential for recall or social desirability bias, particularly in reporting exclusive breastfeeding behaviors. Third, the study was conducted in a single tertiary hospital in Dhaka, which may limit generalizability to rural areas or private‐sector populations. Finally, the breastfeeding practice score, while predictive, may partially overlap with the EBF outcome, raising concerns about conceptual redundancy. Future studies should refine measurement tools to ensure independent constructs.

Despite these limitations, the findings offer important implications for policy and practice. Interventions should prioritize young mothers and those with lower educational attainment, as these groups are at higher risk of suboptimal breastfeeding. Integrating breastfeeding counseling into antenatal and postnatal care, scaling up community health worker follow‐up, and fostering peer‐support networks may improve outcomes. Workplace policies, particularly in labor‐intensive industries such as garment factories, should be strengthened to provide maternity leave, lactation breaks, and breastfeeding‐friendly environments.

Government and non‐governmental organizations can build on the relatively high EBF rates observed in urban hospital settings by expanding breastfeeding promotion programs to rural and underserved communities. Mass media campaigns, regulation of breastmilk substitute marketing, and culturally sensitive educational initiatives should be sustained to reinforce positive breastfeeding practices across diverse sociodemographic groups.

Future research should adopt longitudinal designs to examine the continuity of breastfeeding practices beyond the first year of life and to better understand causal pathways. The dichotomous practice‐score classification may have limited sensitivity; future studies could adopt a three‐tier scale (poor, moderate, good) to capture more nuanced differences. Investigating the role of workplace environments, healthcare provider counseling quality, and family support systems will be essential for designing holistic interventions. Moreover, qualitative research exploring mothers' perceptions of barriers and facilitators could provide nuanced insights into socio‐cultural influences on breastfeeding in Bangladesh.

## 5 Conclusion

The prevalence of exclusive breastfeeding (EBF) among the studied mothers was high (80.4%), reflecting encouraging progress compared to national averages. However, several sociodemographic factors influenced EBF practices. Younger mothers (≤ 26 years) and those with only primary education were less likely to exclusively breastfeed, while higher breastfeeding practice scores were strongly associated with EBF. Other characteristics, such as infant age, birth order, father's education, occupation, place of delivery, and infant sex, did not show independent associations after adjustment. These findings highlight the need for targeted interventions that strengthen breastfeeding knowledge and support, particularly among younger and less‐educated mothers. Workplace accommodations for breastfeeding, especially in labour‐intensive industries, remain essential to sustain EBF. Strengthening community‐based counseling and health system support can also enhance mothers' confidence and ability to practice exclusive breastfeeding. By addressing these factors through coordinated public health policies and programs, Bangladesh can further improve EBF rates, reduce infant morbidity and mortality, and accelerate progress toward achieving Sustainable Development Goal 3 (SDG‐3).

## Author Contributions

**Dilshad Binta Zaman Disha:** conceptualization (equal), data curation (equal), investigation (equal), methodology (equal), writing – original draft (equal). **Md. Shamsur Rahman:** conceptualization (equal), project administration (equal), supervision (equal), validation (equal), writing – review and editing (equal). **Munir Ibn Mahin:** formal analysis (equal), software (lead), visualization (equal), writing – original draft (equal).

## Funding

The authors have nothing to report.

## Conflicts of Interest

The authors declare no conflicts of interest.

## Data Availability

The data that support the findings of this study are available on request from the corresponding author.
